# Tumor-promoting effect and tumor immunity of SRSFs

**DOI:** 10.3389/fcell.2025.1527309

**Published:** 2025-03-10

**Authors:** Shuai Zhang, Yongxi Zhang, Sijia Feng, Miaomiao Han, Zixi Wang, Dan Qiao, Jiaqi Tian, Lan Wang, Baoshun Du, Zheying Zhang, Jiateng Zhong

**Affiliations:** ^1^ Department of Oncology, The Third Affiliated Hospital of Xinxiang Medical University, Xinxiang, China; ^2^ Department of Pathology, The First Affiliated Hospital of Xinxiang Medical University, Xinxiang, China; ^3^ Department of Pathology, School of Basic Medical Sciences, Xinxiang Medical University, Xinxiang, China; ^4^ Department of Pathology, The Third Affiliated Hospital of Xinxiang Medical University, Xinxiang, China; ^5^ Second Department of Neurosurgery, Xinxiang Central Hospital, Xinxiang, China; ^6^ Henan Province Engineering Technology Research Center of Tumor diagnostic biomarkers and RNA interference drugs, The Third Affiliated Hospital of Xinxiang Medical University, Xinxiang, China

**Keywords:** alternative splicing (AS), SRSF, pre-mRNA, tumor, immune

## Abstract

Serine/arginine-rich splicing factors (SRSFs) are a family of 12 RNA-binding proteins crucial for the precursor messenger RNA (pre-mRNA) splicing. SRSFs are involved in RNA metabolism events such as transcription, translation, and nonsense decay during the shuttle between the nucleus and cytoplasm, which are important components of genome diversity and cell viability. SRs recognize splicing elements on pre-mRNA and recruit the spliceosome to regulate splicing. In tumors, aberrant expression of SRSFs leads to aberrant splicing of RNA, affecting the proliferation, migration, and anti-apoptotic ability of tumor cells, highlighting the therapeutic potential of targeted SRSFs for the treatment of diseases. The body’s immune system is closely related to the occurrence and development of tumor, and SRSFs can affect the function of immune cells in the tumor microenvironment by regulating the alternative splicing of tumor immune-related genes. We review the important role of SRSFs-induced aberrant gene expression in a variety of tumors and the immune system, and prospect the application of SRSFs in tumor. We hope that this review will inform future treatment of the disease.

## 1 Introduction

**FIGURE 1:**
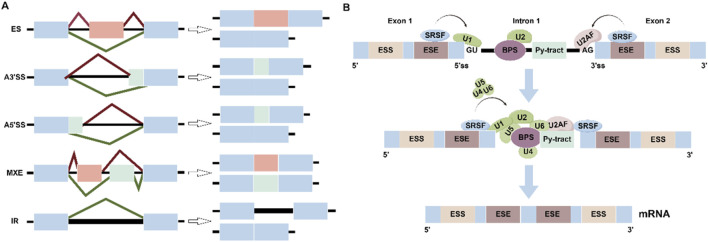
**(A)** Schematic depiction of constitutive splicing and five modes of alternative splicing: exons skipping (ES), alternative 5′ splice site (A5′SS), alternative 3′ splice sites (A3′SS), intron retention (IR) and mutually; exclusive exons (MXE). **(B)** Graphical representation of the stepwise assembly of spliceosomal complexes. SRSFs bind to ESEs to promote the recruitment of U1 snRNP and U2AF1, U2 snRNP and U4/U5/U6 tri-snRNP, lead to spliceosome activation and formation of the mature Mrna.

Alternative pre-mRNA splicing (AS) is an important mechanism for the processing of precursor messenger RNA (pre-mRNA) during gene expression, which greatly enriches the transcriptome pool and promotes transcriptome and proteome diversity (Wilkinson et al., 2020; Zhang Y. et al., 2021). AS is a critical step in higher eukaryotes and is tightly regulated in different tissues, stages of differentiation, and important cellular pathways. In higher eukaryotes, AS is an indispensable process in the mRNA maturation process, which excites introns and ligates exons. AS selectively determines the inclusion or exclusion of exons, and can produce multiple mRNA isoforms based on one pre-mRNA (David and Manley, 2010; Kalsotra and Cooper, 2011). It is estimated that more than 95% of human genes are expressed in favor of AS, and different AS patterns have been identified, including exon skipping (ES), selective 5′splice site (A5′ SS), selective 3′splice site (A3′ SS), Intron retention (IR) and mutexclusive exons (MXE) (Figure 1A).

Pre-mRNA splicing relies on the binding of spliceosomes and some RNA-binding proteins (RBPs) to pre-mRNA cis-acting elements, involving a range of RNA-RNA, RNA-protein, and protein-protein interactions (Lee and Rio, 2015; Sahebi et al., 2016; Savisaar and Hurst, 2016). However, splicing regulatory elements are also present, especially exons, including enhancers or silencers (exon or intron splice enhancers (ESE or ISE) or silencers (ISS or ESS)) located in the intron and exon regions of genes, which regulate splicing by binding to the corresponding trans-acting factors. Serine/arginine-rich splicing factors (SRSFs) are important trans-acting factors that regulate almost every step of AS (Sanford et al., 2004; Long and Caceres, 2009; Manley and Krainer, 2010; Sahebi et al., 2016).

The SR proteins, which are serine/Arginine-rich splicing factors, are a class of RNA-binding proteins (RBPs) consisting of 12 members (SRSF 1–12), the first identified SR proteins are SRSF 1 (formerly known as SF 2/ASF) and SRSF 2 (formerly known as SC 35) (Li and Manley, 2005; Karni et al., 2007; Das and Krainer, 2014). Most SRs are located only within the nucleus, but some SRs (e.g., SRSF 1, SRSF 3, SRSF4, SRSF6, SRSF7, and SRSF 10) can travel between the nucleus and the cytoplasm. SRSF proteins typically consist of one or two RNA recognition motif (RRM) domains at the C-terminus and arginine/serine (RS)-rich regions at the N-terminus. The RRM domain can specifically bind RNA, while the RS region is involved in protein-protein interactions as well as binding to other components of the spliceosome (Aubol et al., 2013).

SR proteins are considered to be essential splicing factors for pre-mRNA splicing, and SR proteins can recognize specific sequences on pre-mRNA, promote the assembly of spliceosomes, and guide the selection of splice sites (Cowper et al., 2001; Zhou and Fu, 2013; Jeong, 2017; Krchnakova et al., 2019; Li D. et al., 2023). Different SR proteins may have different expression levels and activity in different tissues and cell types, affecting the specificity of splicing. They can regulate the production of different splice isomers to suit specific physiological needs. In addition, SR proteins can interact with other splicing factors, RNA-binding proteins, and snRNAs to participate in the functional regulation of spliceosomes. This interaction coordinates the various steps in the splicing process, ensuring accuracy and efficiency of splicing (Ghosh and Adams, 2011; Busch and Hertel, 2012; Shen and Mattox, 2012; Howard and Sanford, 2015). Since SRs play a key role in transcription and translation, aberrant regulation of SRs can severely disrupt the stability of DNA and the normal expression pattern of proteins, which in turn leads to abnormal biological functions (Scotti and Swanson, 2016). In recent years, the role of SRSF as oncoproteins in various tumor types, such as lung, colon, breast and pancreatic cancers, has been widely studied. SRSF plays an important role in tumor immunity, mainly because of its core regulatory function in gene splicing, which can directly affect the biological behavior of tumor cells, immune escape mechanism and the formation of tumor microenvironment, providing a potential target for the treatment of tumor immunity (Juarez et al., 2022; Dias et al., 2024; Lee et al., 2024). The role of SRSF in tumorigenesis and development is summarized here. We also discussed the role of SRSFs in the immune system.

## 2 The role of SRSF in alternative splicing

SRSF (Serine/Arginine-Rich Splicing Factors) is a kind of proteins that play a key role in RNA splicing. SRSF plays an important role in the RNA splicing process (Haynes and Iakoucheva, 2006). They are able to recognize specific RNA sequences, bind to pre-mRNAs, and participate in regulating the inclusion or exclusion of exons, thereby influencing the diversity of gene expression. SRSF members may have specific functions in different tissues and cell types. Its dysfunction may be associated with the occurrence and development of a variety of diseases (Haynes and Iakoucheva, 2006; Das et al., 2007; Twyffels et al., 2011; Will and Luhrmann, 2011; Shi, 2017).

SRSF is mainly composed of two parts: the C-terminal RRM (RNA Recognition Motif) and the N-terminal RS domain (Arginine/Serine-rich domain). The C-terminal RRM is the primary region where the SRSF interacts with the RNA substrate. It has a specific sequence and structure that is able to recognize and bind specific RNA sequences. Differences in RRM sequences are a key factor in the different substrate selectivity of each SRSF(Clery et al., 2021). This difference allows different SRSFs to recognize and bind to different RNA substrates, thus play different roles in the RNA splicing process (Cho et al., 2011a; Cho et al., 2011b; Shimoni-Sebag et al., 2013). The N-terminal RS domain is a sequence of amino acids rich in arginine and serine, which forms the surface of protein interactions. This region is able to interact with a variety of other proteins, including other components in the spliceosome, kinases, phosphatases, etc. The RS domain is also the primary site of post-translational modifications (PTMs). These modifications include phosphorylation, methylation, etc., which are essential for regulating the function and activity of SRSF(Edmond et al., 2011; Xiao et al., 2016; Khan et al., 2024).

The structural characteristics of SRSF (particularly the RS domain at the N-terminus) are critical to its function. The unique RS domain is an important structure that distinguishes SRs from other RNA-binding proteins. SRs, as a ubiquitous splicing factor, play an important role in alternative splicing (Erkelenz et al., 2013). In the splicing reaction, SRSF binds to exon splice enhancers (ESEs) to recruit small ribonucleoprotein (snRNP) and its cofactors to the splice site, and SR then promotes the binding of the U2 snRNA to the branching area of the pre-mRNA to form a complex, which then bridges these splice site recognitions (Cho et al., 2011a; Busch and Hertel, 2012; Erkelenz et al., 2013). In the initial stage of spliceosome assembly, SRSFs bind ESE at exons to recruit U1 snRNPs and U2AF to facilitate spliceosome assembly and exon definition, and subsequently, SRSFs promote the recruitment of U4/U5/U6 snRNPs to form catalytically active complexes through rearrangements between snRNPs to complete intron excision and exon junction, in addition, the location of SRSF at the pre-mRNA site affects the splicing results (Michlewski et al., 2008; Jobbins et al., 2022) (Figure 1B). For example, exon-bound SRSF act as enhancers, but intron-bound SRSFs act as repressors. Therefore, the location of the SRs-pre-mRNA interaction influences the selection of splice sites and the assembly of spliceosomes (Erkelenz et al., 2013).

## 3 The role of SRSF in cancer

**FIGURE 2:**
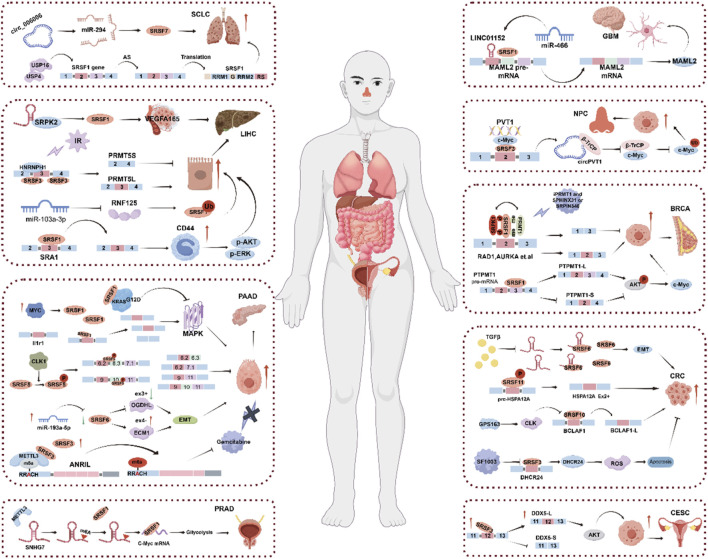
The role of SRSF in cancer.

Almost all tumors have a complex pathogenesis with a variety of factors involved. SRSF (serine/arginine-enriched splicing factor) family members play an important role in the occurrence and development of a variety of cancers (Cowper et al., 2001; Long and Caceres, 2009; Zhang Y. et al., 2021). SRSF is involved in the alternative splicing process of pre-mRNA by regulating the production of splice isoforms of specific genes, so the abnormal regulation of SRSFs can seriously disrupt the stability of DNA and the normal expression pattern of proteins, resulting in abnormal biological functions, and then participate in the occurrence and development of cancer. SRSF can bind to specific RNA sequences and affect the post-transcriptional regulation of genes, including mRNA stability, translation efficiency, etc. This may lead to changes in the expression level of tumor-related genes, which in turn affects the tumor progression (Oltean and Bates, 2014; Shilo et al., 2015; Scotti and Swanson, 2016; Obeng et al., 2019; Che and Fu, 2020). This suggests that SRSF splicing factors play an important role in tumor (Figure 2; Table 1).

**TABLE 1:** Dysregulated SRSF expression in cancer.

Cancer type	SRs	Target gene	Biobehavioral functions	References
Colorectal cancer (CRC)	SRSF3、SRSF6、SRSF7、SRSF9、SRSF10、SRSF11	SRF、B7-H3、ArhGAP30/Ace-p53、LINC 01133、LINC 01123、EEF1D、GPS 167、BCLAF1-L、HSPA12A	SRSF3 binding SRF regulates VEGF to promote CRC proliferation; SRSF3 binds B7-H3 mRNA to promote the CRC progression; SRSF3 promotes the CRC progression via the ArhGAP30/Ace-p53 signaling pathway; SRSF6 is reduced in binding to LINC 01133 to promote CRC progression; LINC 01123 promotes CRC progression via interacting with SRSF7; SRSF9 upregulates EEF1D to promote the CRC proliferation and metastasis; GPS 167 leads SRSF10 to promote CRC progression; SRSF10 promotes the generation of BCLAF1-L splice variants to promote the CRC progression; SRSF11 regulates the splicing and self-phosphorylation status of HSPA12A to promote the CRC progression	Manetti et al. (2011), Zhou et al. (2014), Kong et al. (2016), Wang et al. (2020a), Sohail et al. (2021), Zhang et al. (2021a), Chen et al. (2022), Liu et al. (2022e), Pan et al. (2022), Wang et al. (2024)
Pancreatic cancer (PAAD)	SRSF1、SRSF3、SRSF5、SRSF6	IL1R1、CLK1、miR-193a-5p、lncRNA ANRIL	SRSF1 regulates MAPK signaling pathway to promote PAAD progression; SRSF5 promotes the proliferation of PAAD cancer cells under CLK1 phosphorylation; miR-193a-5p targets SRSF6 to promote PAAD progression; SRSF3 regulates lncRNA ANRIL and m6A modification to improve drug resistance in cancer cells	Li et al. (2020), Chen et al. (2021b), Wang et al. (2022c), Wan et al. (2023)
Hepatocellular carcinoma (HCC)	SRSF1、SRSF2、SRSF3、SRSF9、SRSF10	MALAT1、RNF125、LINC 01446、SRA1、HLTF、ANRIL、PPM1G、TUG1、AXIN1、CDC25A	MALAT1 increased the SRSF1 to promote glycolysis and the HCC progression; SRSF1 inhibitors (RNF125) inhibited HCC progression; LINC01446 activates SRPK2/SRSF1/VEGFA to promote HCC progression; SRSF1 activates SRA1 to promote the HCC progression; SRSF1 interacts with HLTF to activate ERK/MAPK signaling pathway to promote HCC progression; ANRIL modulates the miR-199a-5p/SRSF1 axis to promote HCC progression; PPM1G regulates the phosphorylation of SRSF3 and promotes the progression of HCC; TUG1 increased the SRSF9 expression to promote the proliferation and migration of HCC cells; The interaction between SRSF9 and AXIN1 inhibits HCC progression; SRSF10 dephosphorylation of CDC25A promotes the HCC progression	Chen et al. (2021a), Lei et al. (2021), Liu et al. (2022c), Liu et al. (2022d), Feng et al. (2023), Xu et al. (2023b), Wu et al. (2024), Zhang et al. (2024a), Zhang et al. (2024b)
breast cancer (BRCA)	SRSF1、SRSF2、SRSF3	PTPMT1、PLOD2、GRa、RACK1、TDP-43、PRMT1	SRSF1 regulates PTPMT1 alternative splicing to promote breast cancer progression; SRSF2 regulates PLOD2 alternative splicing to promote breast cancer progression; SRSF3 promotes the expression of GRa and RACK1 to promote breast cancer progression; Loss of TDP43 inhibits breast cancer progression in coordination with SRSF3; Targeting PRMT1-mediated SRSF1 methylation inhibits breast cancer progression	Ke et al. (2018), Sheng et al. (2018), Buoso et al. (2019), Du et al. (2021), Li et al. (2023b)
Cervical cancer (CESC)	SRSF1、SRSF3、SRSF9	DDX5、MIR155HG、MicroRNA-802、miR-1	SRSF3 regulates DDX5 alternative splicing to promote Cervical cancer; MIR155HG binds SRSF1 to inhibit Cervical cancer progression; MicroRNA-802 and miR-1 targets SRSF9 to inhibit Cervical cancer progression	Zhang et al. (2019), Shen et al. (2020), Che et al. (2023)
Prostate cancer (PRAD)	SRSF1	SNHG7	SNHG7 promotes glycolysis via the SRSF1/c-Myc axis to promote the PRAD progression	Liu et al. (2022b)
Small cell lung cancer (SCLC)	SRSF1、SRSF2、SRSF7	USP15、USP4、VEGFR1、Circ_006006	USP15 and USP4 regulate the SRSF1 alternative splicing to promote the proliferation of SCLC cancer cells; SRSF2 controls VEGFR1 precursor mRNA to promote SCLC progression; Circ_006006 upregulates SRSF7 to promote SCLC progression	Abou Faycal et al. (2019), Das et al. (2022), Xu et al. (2022)
Glioblastoma (GBM)	SRSF1、SRSF7、SRSF9	LINC01152、CDK1	LINC01152 regulates SRSF1 alternative splicing to promote GBM progression; SRSF7 promotes the GBM progression through m6A modification; SRSF9 combined with CDK1 gene to promote the GBM progression	Wu et al. (2021), Cun et al. (2023), Luo et al. (2024)
nasopharyngeal carcinoma (NPC)	SRSF1、SRSF3、SRSF10	circPVT1、AMOTL1、circCAMSAP1	SRSF1-mediated circPVT1 promotes the NPC progression; SRSF3 regulates AMOTL1 to promote the NPC progression; c-Myc synergistically promotes the carcinogenicity of circCAMSAP1 with the splicing factor SRSF10	Mo et al. (2022), Wang et al. (2022b), Xu et al. (2023a)

As an oncoprotein, SRSF has been extensively studied in colorectal cancer, pancreatic cancer, hepatocellular carcinoma, small cell lung cancer, glioblastoma, nasopharyngeal carcinoma, and reproductive system diseases.

### 3.1 Colorectal cancer

Colorectal cancer (CRC), which originates from the colorectal epithelium and typically includes colon and rectal cancers, is one of the malignant tumors with a high incidence rate worldwide with colorectal adenocarcinoma being the most common, accounting for about 95% of all colorectal malignant tumors (Dekker et al., 2019). SRSFs play an important role in colorectal cancer, influencing gene expression and function by regulating mRNA precursor splicing, thereby contributing to colorectal carcinogenesis, development, and progression.

In colorectal cancer, SRSF splicing factors have the ability to control the splicing of several genes that are directly linked to carcinogenesis and development. For example, SRSF3 affects SRF mRNA splicing by directly binding to a specific motif (“CAUC”) in exon 6 of SRF (serum response factor), which then regulates the expression of downstream genes, such as VEGF (vascular endothelial growth factor), thereby enhancing the proliferation and migration of tumor cells and angiogenesis (Chen et al., 2022). Furthermore, SRSF6 was found to be significantly upregulated in colorectal cancer tissues and associated with tumor prognosis. As an oncogene, SRSF 6 promotes the growth, migration, and invasiveness of colorectal cancer by directly binding to its motifs in exon 23 and controlling the erroneous splicing of ZO-1 (Wan et al., 2019).

Aberrant expression or altered function of SRSF splicing factors can significantly affect the biological behavior of colorectal cancer cells. For example, LINC 01133, which is downregulated by TGFβ, interacts directly with the splicing factor SRSF6 and SRSF6 binding to LINC01133 is reduced, encouraging EMT and CRC cell metastasis (Kong et al., 2016). Similarly, SRSF3 affected the proliferation, migration, and survival of CRC cells through the ArhGAP30/Ace-p53 signaling pathway; SRSF9 upregulated EEF1D (eukaryotic translation elongation factor 1D) to promote CRC proliferation and metastasis (Wang J. L. et al., 2020; Wang et al., 2024). GPS 167 was able to promote the activity of the CLK splicing factor, which in turn reprogrammed the tumorigenic activity of SRSF10 in CRC cells (Sohail et al., 2021). Elevated SRSF10 expression promotes the production of BCLAF1-L splice variants, which affects tumor cell growth and migration (Zhou et al., 2014). SRSF11 promotes CRC metastasis by regulating the splicing of HSPA12A and its own phosphorylation status (Pan et al., 2022).

SRSF splicing factors are associated with colorectal cancer development and prognosis. For example, SRSF3 significantly inhibited the expression of B7-H3 in CRC cells, and the high expression of B7-H3 was associated with poor prognosis of the patients. SRSF3 directly binds to B7-H3 mRNA and participates in its splicing process, which further suggests the potential role of SRSF splicing factors in the assessment of prognosis (Zhang C. et al., 2021).

Given the important role of SRSF splicing factors in colorectal cancer, they are considered potential therapeutic targets. By developing targeted drugs or inhibitors against SRSF splicing factors, the normal biological behavior of tumor cells can be interfered, thereby inhibiting tumor growth and spread. For example, studies have reported the antitumor activity of SFI003, a targeted inhibitor against SRSF3, in colorectal cancer, where SFI003 induced apoptosis in CRC cells through the SRSF3/DHCR24/ROS axis, and exhibited potent antitumor efficacy both *in vitro* and *in vivo* (Zhang et al., 2022). Similarly, it has been shown that inhibiting SRSF9 can enhance the sensitivity of CRC to ferroptosis, suggesting its probability as a potential therapeutic target (Wang X. et al., 2022). SRSF splicing factors play an important role in colorectal cancer, and they influence the biological behavior of colorectal cancer by regulating the splicing process of related genes. In the future, with the in-depth study of the specific mechanism of SRSF splicing factors in colorectal cancer and the development of targeted drugs or inhibitors against these splicing factors, new ideas and methods will be provided for the diagnosis and treatment of colorectal cancer.

### 3.2 Pancreatic cancer

Pancreatic cancer (PAAD) is a malignant tumor originating from pancreatic ductal epithelium and acinar cells, which is called “the king of cancers” by the medical field. Pancreatic cancer is a highly malignant tumor with complex and diverse pathogenic causes but the early symptoms are subtle, making diagnosis and treatment challenging (Vincent et al., 2011). In order to reduce the morbidity and mortality of pancreatic cancer, we need to strengthen the research on its etiology and pathogenesis. In the study of pancreatic cancer, SRSF family members have been found to be related to the occurrence, development, and prognosis of pancreatic cancer.

The following is a detailed description of the association between SRSF and pancreatic cancer: SRSF1 has been demonstrated to regulate and enhance the stability of interleukin 1 receptor type 1 (IL1R1) mRNA via alternative splicing, which increases IL1R1-L expression. This upregulation further activates the MAPK signaling pathway, an important signaling pathway in pancreatic carcinogenesis, when KRASG12D activating mutations can promote ubiquitination degradation of SRSF1, which in turn alleviates the activation of MAPK signaling and maintains pancreatic cell homeostasis. In pancreatic cells overexpressing KRASG12D, SRSF1 is regulated by a negative feedback mechanism (Wan et al., 2023). When MYC signaling is hyperactive, it can remove this negative feedback inhibition, resulting in increasing SRSF1 expression and further speeding up pancreatic cancer. The CLK1-SRSF5 axis is critical to the development of pancreatic cancer. CLK1, a phosphorylated kinase and selective splicing factor, is able to participate in a wide range of phosphorylation regulation of selective splicing-associated proteins, in particular, the SRSF5-ser-250 site. SRSF5 inhibits METTL14 exon 10 jumping in response to CLK1 phosphorylation, while promoting cell cycle protein L2 exon 6.3 jumping. In addition, aberrant METTL14 exon 10 jumping enhanced N6-methyladenosine modification levels and metastasis, whereas aberrant Cyclin L2 exon 6.3 promoted the proliferation of PDAC cells (Chen S. et al., 2021). It has been shown that miRNAs play an important role in targeting SRSF splicing factors in pancreatic cancer metastasis. MiR-193 a-5p regulates oxoglutarate dehydrogenase-like (OGDHL) and extracellular matrix protein 1 (ECM 1) alternative splicing by targeting SRSF 6, which triggers epithelial-mesenchymal transition (EMT) and consequently enhances pancreatic cancer progression (Li et al., 2020). Meanwhile, SRSF3 boosted the resistance of pancreatic cancer cells to chemotherapeutic drugs (e.g., gemcitabine) by modulating lncRNA ANRIL splicing and N6-methyladenosine (m6A) modification (Wang Z. W. et al., 2022). This resistance mechanism makes the treatment of pancreatic cancer more difficult. Due to the important role of SRSF splicing factors in pancreatic cancer development, they could be prospective targets for pancreatic cancer therapy. Inhibiting the function or expression of these splicing factors may assist in slowing the progression of pancreatic cancer and improve patients’ prognoses. SRSF splicing factors play an important role in pancreatic cancer, affecting pancreatic cancer development and progression by regulating RNA splicing and signaling pathways. Future research will reveal the precise mechanism of these splicing factors and investigate their potential as therapeutic targets for pancreatic cancer.

### 3.3 Hepatocellular carcinoma

Hepatocellular carcinoma (HCC), one of the deadliest malignant cancers in the world, has been a hot research topic for its complex molecular mechanisms (Wang and Deng, 2023). NcRNA plays an important role in Hepatocellular carcinoma by regulating the SRSFs biological activity. For example, LINC01446, derived from LTR retrotransposon, is mainly activated in HCC and correlates with the proliferation state of HCC, and HCC patients with higher expression of LINC01446 have a shorter overall survival time. LINC01446 binds serine/arginine protein kinase 2 (SRPK2) to activate its downstream target serine/arginine splice factor 1 (SRSF1), and activation of the SRPK2-SRSF1 axis increases the splicing and expression of the VEGF isoform A165 (VEGFA165) and thus contributes to the HCC progression (Wu et al., 2024). lncRNA MALAT1 acts as a proto-oncogene by activating the Wnt pathway and inducing the oncogenic splicing factor SRSF1. MALAT1-induced SRSF1 regulates SRSF1 splicing targets, enhances the production of anti-apoptotic splice isoforms and activates the mTOR pathway by regulating the variable splicing of S6K1. Inhibition of SRSF1 expression or mTOR activity may abolish MALAT’s oncogenic characteristics. In addition, the biological activities of SRSFs have been associated with poor prognosis in HCC patients (Hu et al., 2016). For example, the E3 ubiquitin ligase, RNF125, was dramatically downregulated in HCC tumor tissues, and experimental results indicate that RNF125 interacts with SRSF1. RNF125 enhances proteasome-mediated SRSF1 degradation, thereby prevents HCC progression through inhibition of the ERK signaling pathway. RNF125 was identified as a downstream target of miR-103a-3p, which blocked RNF125 binding to SRSF1(Feng et al., 2023).

Steroid receptor RNA activator 1 (SRA1) has been described as a novel transcriptional co-activator that affects cancer cell migration. It has been discovered that SRSF1 binds to pre-mRNA of SRA1 located in the region surrounding the exon 3′splice site, generating the long isoform SRA1-L. Overexpression of either SRSF1 or SRA1-L promotes migration and invasion of hepatocellular carcinoma cells by enhancing the expression of CD44 (Lei et al., 2021). Protein arginine methyltransferase 5 (PRMT5) is an epigenetic regulator, and we found that SRSF3 and HNRNPH1 competitively bind to PRMT5 pre-mRNAs located in the region around the 3′- splice site on intron 2 and selective 3′- splice site on exon 4, generating the long isoform of PRMT5L to promote hepatocellular carcinoma progression. However, IR (Ionizing Radiation)-induced SRSF3 downregulation resulted in elevated levels of the splice isoform PRMT5S to inhibit hepatocellular carcinoma progression (Wen et al., 2022). Subsequent research will elucidate the precise mechanism of these splicing factors, providing targets for the therapy of hepatocellular carcinoma.

### 3.4 Diseases of the reproductive system

#### 3.4.1 Breast cancer

One of the most prevalent malignant tumors in women, breast cancer (BRCA) has many different and intricate origins. Breast cancer is a malignant tumor that requires high attention. It can be prevented and treated more effectively if its causes, symptoms, manifestations, diagnostic techniques, and prognosis are understood (Veronesi et al., 2005; Jokar et al., 2021). SRSF splicing factors can affect breast cancer-related gene expression patterns by regulating the alternative splicing of specific genes. This regulation can impact the biological activities of breast cancer cells such as proliferation, migration, invasion, and apoptosis.

In breast cancer tissues, high expression of SRSF1 (sometimes referred to as SF2/ASF) represents a significant unfavorable prognostic predictor. It accelerates breast cancer progression by regulating exon 3 splicing of PTPMT1 (a phosphatase), altering the function of PTPMT1 and then activating the AKT/C-MYC signaling pathway, which promotes breast cancer cell proliferation and migration, and inhibits apoptosis (Du et al., 2021). Similarly, SRSF2 has been demonstrated to influence the malignant growth of human breast cancer by controlling the alternative splicing of PLOD2. Cortisol induces the expression of SRSF3, which then promotes splicing changes in the glucocorticoid receptor GRa and the expression of RACK1 (receptor binding protein 1). Thus, SRSF3 can promote the migration and invasion of breast cancer cells (Buoso et al., 2019). Studies have shown that TAR DNA binding protein 43 (TDP-43) is highly expressed in triple-negative breast cancer (TNBC) and correlates with poor patient prognosis. TDP43 forms a complex with SRSF3 and they co-regulates gene-specific splicing events, which in turn affect the proliferation and migration ability of TNBC cells. This reveals the synergistic mechanism of TDP43 and SRSF3 in TNBC and provides new targets and ideas for the treatment of TNBC (Ke et al., 2018).

PRMT1 (protein arginine methyltransferase 1) is overexpressed in breast cancer, and PRMT1-mediated SRSF1 methylation and SRPK1-mediated SRSF1 phosphorylation are interdependent; PRMT1 enhances the phosphorylation, RNA-binding capacity, and exon splicing activity of SRSF1 through methylation of SRSF1, and this interaction enhances the oncogenic potential of SRSF1. Notably, the inhibitor of PRMT1 (iPRMT1) was able to inhibit breast cancer cell growth, and the iPRMT1 was even more effective when combined with inhibitors targeting SRSF1 phosphorylation (SPHINX31 and SRPIN340), suggesting the potential value of combination therapy (Li W. J. et al., 2023). Subsequent studies could further explore the upstream and downstream signaling pathways of SRSF splicing factors, as well as their interactions with other cancer-related genes to discover more effective therapeutic approaches that could contribute to better prevention and treatment of breast cancer.

#### 3.4.2 Cervical cancer

Cervical cancer (CESC) is a malignant tumor that poses a serious threat to women’s health (Burd, 2003). The role of SRSF (serine/arginine-rich splicing factor) family members in cervical cancer and their complex relationships with variable splicing, signaling pathways, and immune escape provide new perspectives on molecular mechanism studies and the development of therapeutic strategies for cervical cancer. It has been shown that the high expression of SRSF3 in cervical cancer promotes the generation of the oncogenic spliceosomes DDX5-L and inhibits the generation of the suppressor spliceosome DDX5-S. This splicing regulatory mechanism enhances the proliferation, migration, and invasion of cervical cancer cells and promotes the malignant progression of cervical cancer by up-regulating the AKT signaling pathway. In addition, the conduction of this oncogenic signaling pathway can be blocked by direct inhibition of SRSF3(Che et al., 2023). NcRNAs play an important role in the development of cervical cancer through the regulation of SRSF family members. For example, by binding to SRSF1, MIR155HG in DElncRNA enhances the inhibitory effect on SRSF1 to slow down the cervical cancer progression (Shen et al., 2020). MicroRNA-802 and miR-1 inhibited cervical cancer proliferation and induced apoptosis by targeting SRSF9 (Zhang et al., 2019). The study on SRSF splicing factor in cervical cancer provides new ideas and methods for the treatment of cervical cancer. With the deepening of research and the continuous development of technology, it is believed that more therapeutic methods targeting SRSFs will be applied to the clinic in the future, which will bring better therapeutic effects and life quality for cervical cancer patients.

#### 3.4.3 Prostate cancer

The most frequent malignant tumor of the male genitourinary system is prostate cancer (PRAD), an epithelial malignant tumor that develops in the prostate gland. SRSFs may affect prostate cancer development by regulating the splicing process of key genes in prostate cancer (Sette, 2013). There are relatively few studies on specific SRSF splicing factors in prostate cancer, but several of them have revealed their potential roles in prostate cancer. Prostate cancer cells tend to have high glycolytic activity and tend to produce energy through glycolysis even in the presence of sufficient oxygen (Warburg effect), which provides energy and intermediates needed for rapid tumor proliferation and biosynthesis. One such example is the m6A modification of SNHG7 which is mediated by methyltransferase-like 3 (METTL3), which greatly increases its stability. It has been established that the small nuclear kernel RNA host gene 7 (SNHG7) has an oncogenic function in prostate cancer. SNHG7 contributes to the development of prostate cancer by promoting glycolysis via the SRSF1/c-Myc axis in two prostate cancer cell lines, PC-3 and DU-145 (Liu et al., 2022b). Since the information above suggests that SRSF is crucial for the development of prostate cancer, it is possible to prevent the growth and proliferation of tumors by reducing the activity or expression level of SRSF splicing factor. However, targeted therapeutic drugs have not yet found widespread clinical usage and are still in the research and development phase when it comes to the SRSF splicing factor. Future studies will advance the use of the SRSF splicing factor in the treatment of prostate cancer by elucidating its mechanism in greater detail.

### 3.5 Small cell lung cancer

A form of lung cancer that starts in the glands or bronchial mucosa, small-cell lung carcinoma (SCLC) is known for its early onset of distant metastases and unfavorable prognosis. The malfunction and aberrant expression of SRSF family members are especially significant in small-cell lung cancer, a tumor form that is extremely aggressive and difficult to treat (Ko et al., 2021; Yin et al., 2022). SRSF splicing factors have the ability to influence a number of biological processes in SCLC cells, including cell division, proliferation, and apoptosis. Research has indicated that SRSF1 plays a significant role in SCLC tumorigenicity and may control tumor progression by influencing DNA damage and chemotherapy sensitivity. This suggests that SRSF1 promotes SCLC malignant progression by regulating gene splicing associated with tumor growth, invasion and metastasis (Jiang et al., 2016). Additionally, USP 15 and its homologue (USP 4) promote lung cancer cell proliferation by regulating SRSF1 alternative splicing, so targeting therapy against SRSF1 splicing factor may become a new strategy for SCLC treatment (Das et al., 2022). The goal of preventing tumor development and proliferation can be accomplished by interfering with the normal biological behavior of tumor cells through the inhibition of SRSF splicing factor activity or expression level. According to studies, the signaling network of VEGFR1 pre-mRNA alternative splicing offers a novel approach and possible therapeutic target for the treatment of small-cell lung cancer. The three proteins VEGF165, SOX2, and SRSF2 work together to regulate the alternative splicing of VEGFR1 pre-mRNA to produce the truncated sVEGFR1-i13 receptor. The signaling network is strengthened by upregulating sVEGFR1-i13 receptor in the presence of anti-angiogenic therapy-stimulated upregulation of sVEGFR1-i13 protein levels in squamous lung cancer cells to prevent the progression of small cell lung cancer (Abou Faycal et al., 2019). It has been demonstrated that ncRNAs play significant roles in SCLC development through the regulation of SRSF family members. For example, Circ_006006 increases the expression of SRSF7 (a miR-294 target) through sponging, which further advances the development of SCLC (Xu et al., 2022). In conclusion, SRSF splicing factor is a significant contributor to small cell lung cancer and could be a novel target for future diagnosis and therapy of this disease.

### 3.6 Glioblastoma

Patients with glioblastoma (GBM), a highly aggressive brain tumor, endure excruciating physical and mental pain and their prognosis is not well (Hawly et al., 2024). SRSF3 is often upregulated in clinical glioma specimens, and it has been demonstrated that high SRSF3 is associated with tumor progression and a bad prognosis for glioma patients (Song et al., 2019). GBM staging and poor glioma patient survival are also linked to elevated SRSF9 expression (Liu et al., 2022a). SRSF9 attaches to the CDK1 gene promoter and raises its transcriptional level, which promotes the growth of GBM cells. Both LINC01152 and transcriptional co-activator 2 (MAML2), among others, have been demonstrated to have oncogenic roles in GBM. It has also been demonstrated that LINC01152 is considerably upregulated in tissues and cells and plays a role in the progression of GBM. By recruiting SRSF1 and positively regulating MAML2 in GBM via the sponge miR-466, LINC01152 stimulates the development of GBM (Wu et al., 2021). SRSF7 gene expression is positively correlated with glioblastoma (GBM) cell-specific m6A methylation. SRSF7 promotes GBM cell proliferation and migration, which is mostly reliant on the presence of m6A methyltransferases. Insulin-like growth factor 2 mRNA regulates SRSF7, which in turn regulates the two m6A sites on PDZ-binding kinase (PBK) mRNA. SRSF7 exerts its activity in GBM cells by recognizing insulin-like growth factor 2 mRNA-binding protein 2 (IGF2BP2). Therefore, SRSF7 is a novel m6A regulator promoting GBM progression (Cun et al., 2023). Future research on the precise mechanism of SRSF splicing factor in glioblastoma will shed additional light on the connection between SRSF and glioblastoma and help identify new targets for glioblastoma treatment.

### 3.7 Nasopharyngeal cancer

Nasopharyngeal carcinoma (NPC) is a highly malignant tumor with complex and diverse pathogenesis (Chen et al., 2019). SRSF, as a major mRNA regulator, plays an important role in tumorigenesis and development. Although there are relatively few studies directly targeting the association between nasopharyngeal carcinoma and SRSF, it has been demonstrated that SRSF1-mediated circPVT1 maturation processing positive feedback regulates circPVT1 expression in nasopharyngeal carcinoma, which provides a candidate molecular marker and a potential therapeutic target for the diagnosis of metastatic nasopharyngeal carcinoma. This study provides candidate molecular markers and potential therapeutic targets for the diagnosis of metastatic NPC (Mo et al., 2022). In addition, circCAMSAP1 is highly expressed in nasopharyngeal cancer tissues and promotes the proliferation and nasopharyngeal cancer metastasis. c-MyC interacts with SRSF10 splicing factor and promotes the transcription and reverse splicing of CAMSAP1 pre-mRNA, resulting in positive feedback generated by circCAMSAP1, which leads to nasopharyngeal cancer proliferation and metastasis, and also suggests a role for SRSF in the development and possible treatment of nasopharyngeal cancer (Mo et al., 2022). These studies indicate that SRSF plays an important role in the development, progression, and metastasis of nasopharyngeal carcinoma, and its related pathways may provide new ideas and methods for the treatment of nasopharyngeal carcinoma. However, these studies are still in the laboratory stage, and further clinical studies are needed to verify their effectiveness and safety in the treatment of nasopharyngeal carcinoma.

## 4 The role of SRSF in tumor immunity

**FIGURE 3:**
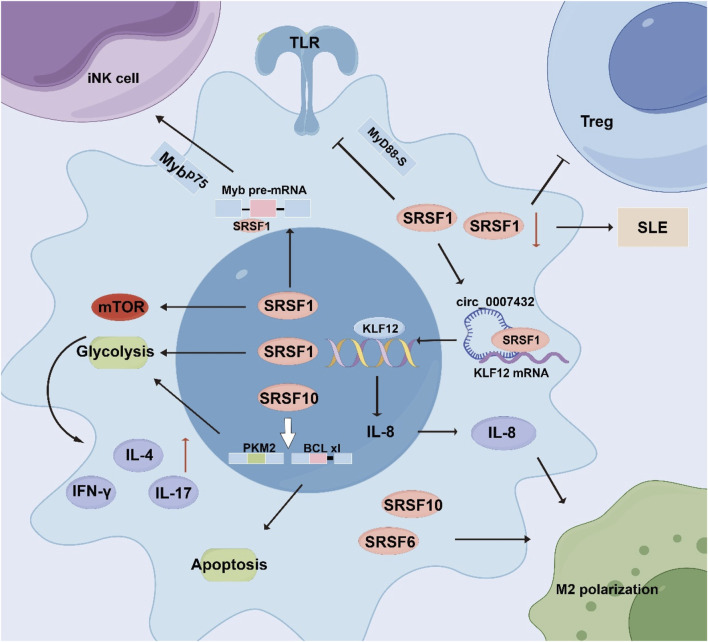
The role of SRSF in tumor immunity.

In the tumor microenvironment, tumor cells stimulate the expression of immune checkpoint molecules by binding to cytokine-secreting immunosuppressive cells, negatively affecting CTLs and leading to T cell exhaustion and immune escape. SRSFs can affect immune cell function in the tumor microenvironment by regulating the alternative splicing of tumor immune-associated genes. SRSF splicing factors play important roles in the development, differentiation, function, and disease development of T cells and related immune cells (Figure 3).

SRSF1 promotes the production of pro-inflammatory cytokines by controlling glycolytic metabolism and mTORC1 activity. SRSF1 can affect the iNKT cell’s development and function by regulating the alternative splicing of the important transcription factor Myb, and can also affect the TLR’s function by regulating the MyD88 alternative splicing. The SRSF1 downregulation will lead to impaired function of Treg cells, leading to severe autoimmune and organ inflammation. Meanwhile, the SRSF1 downregulation is related to SLE; SRSF10 can affect the production of pro-inflammatory cytokines through glycolysis and can also promote the apoptosis of T cells through alternative splicing. In addition, SRSF6 and SRSF10 affect the polarization of M2 macrophages.

### 4.1 T cells

T cells are an essential component of immune system and play a key role in the recognition and clearance of pathogens, tumor cells, etc. T cells recognize antigens through their surface T cell receptors (TCRs), upon antigen stimulation, become activated, proliferate, and differentiate into effector T cells and memory T cells, thereby mediating adaptive immune responses (Siska and Rathmell, 2015; Kishton et al., 2017). SRSF splicing factors play an important role in the T cells progression, and its abnormality is closely related to the development of T cell-related diseases. SRSF splicing factors regulate T cell development and differentiation.

Treg cells, known as regulatory T cells (Treg for short), are a subpopulation of T cells that play an important regulatory role in the immune system (Whiteside, 2018; Goschl et al., 2019). It has been shown that SRSF1 is necessary for the homeostasis and normal function of Treg cells, and Its absence leads to impaired Treg cell function, which in turn triggers severe autoimmunity and organ inflammation. Meanwhile, SRSF1 finely regulates Treg cell function by controlling glycolytic metabolism, mTORC1 activity, and pro-inflammatory cytokine production, which is important for maintaining immune homeostasis (Katsuyama et al., 2019).

SRSF splicing factors are associated with these functions of maintaining homeostasis, promoting proliferation, maintaining cytotoxicity, and antiviral immune responses to CD8 T cells. For example, the splicing factor SRSF1 maintains the normal homeostasis of CD8 T cells, promotes their efficient proliferation, and maintains their cytotoxicity by regulating cell proliferation, MAP kinase signaling, and the IFN (interferon) signaling pathway. In viral infection models, SRSF1 is required for antigen-specific IFN-γ cytokine responses, which is essential for virus clearance (Juarez et al., 2022). SRSF splicing factors play a critical role in the maturation of late-stage thymocytes, especially for CD8 single-positive T-cell fate determination. For example, SRSF1 affects this process by regulating the expression of key transcription factors such as Runx3, revealing an important role for the splicing factor SRSFs in the development of immune system and potentially providing new targets for the treatment of immunodeficiency diseases (Ji et al., 2022).

iNKT cells, known as Invariant Natural Killer T cells, or Constant Natural Killer T cells, are immune cells with specialized functions that incorporate the properties of T cells and natural killer (NK) cells (Qin et al., 2022). SRSF family proteins, as an important class of RNA-binding proteins, play important roles in the development, differentiation and function of iNKT cells. For example, SRSF1 maintains the abundance of Mybp75 (the short isoform of Myb) by regulating the alternative splicing of the important transcription factor Myb, which in turn affects the development and function of iNKT cells. iNKT cell proliferation is reduced and apoptosis is increased when SRSF1 is deficient, as well as TCRα rearrangement and apoptosis of its precursor DP cells are also abnormal, which ultimately leads to iNKT cell function being Impaired (Liu et al., 2021). Mutations or aberrant expression of SRSF splicing factors are closely associated with the development of T cell-related diseases. For example, in T-cell acute lymphoblastic leukemia (T-ALL), mutations or altered expression levels of splicing regulators such as SF3B1 and SRSF2 are thought to play an important role in the development of leukemia (Yoshimi et al., 2019).

TILs (tumor-infiltrating lymphocytes) are a class of immune cells capable of specifically recognizing and attacking tumor cells. TILS play an important role in tumor immunotherapy, as they are capable of highly specific recognition and killing of tumor cells, while reducing damage to normal cells (Lin et al., 2020). SRSF2 is overexpressed in depleted T cells and is involved in the depletion of tumor-infiltrating lymphocytes (TILs). SRSF2 enhances anti-tumor immune responses by affecting H3K27Ac levels of related genes and the recruitment of STAT3, providing a new idea for reversing the depletion of TILs (Wang Z. et al., 2020). Immunotherapeutic strategies targeting SRSF2 or its regulatory pathways may help restore the function of TILs and improve the efficacy of cancer immunotherapy.

### 4.2 Systemic lupus erythematosus (SLE)

Systemic Lupus Erythematosus (SLE) is a complex autoimmune disease characterized by abnormal activation of the immune system, which attacks its own tissues, leading to multi-system and multi-organ damage (Kiriakidou and Ching, 2020). The splicing factor SRSF1, an important RNA-binding protein, plays a key role in the pathogenesis of SLE. It affects the immune response and inflammatory response in SLE by controlling T cell homeostasis and cytokine production. Studies have shown that levels of the splicing factor SRSF1 are abnormally low in T cells from SLE patients and correlate with disease severity. This finding suggests that SRSF1 may be a potential target for SLE treatment (Katsuyama et al., 2020). SRSF1-deficient mice were found to exhibit systemic autoimmunity and lupus nephritis, which may be due to the impaired ability of SRSF1 to inhibit T-cell activation by suppressing mTOR pathway activity and maintaining PTEN expression. Reduced PTEN levels have also been observed in T cells from SLE patients, correlating with reduced SRSF1(Katsuyama et al., 2019). It was shown that estrogen upregulates hsa-miR-10b-5p expression in human T cells, whereas hsa-miR-10b-5p affects T cell function by downregulating SRSF1 protein expression. This finding provides a new perspective for understanding the potential role of estrogen in the pathogenesis of SLE. Therefore, studies targeting SRSF1 may provide new ideas and methods for the diagnosis, treatment and prognostic assessment of SLE (Katsuyama et al., 2019). Further in-depth studies on the specific mechanism of SRSF1’s role in SLE and its interaction with other immune cells and molecules are needed in the future to promote the development of precision medicine and individualised treatment of SLE.

### 4.3 Innate immunity receptors

TLRs (Toll-like receptors) are a class of pattern recognition receptors (PRRs) that play a key role in the innate immune system. Activation of TLR triggers a range of immune responses, including production of pro-inflammatory cytokines, upregulation of co-stimulatory molecules, and maturation and migration of antigen-presenting cells (e.g., dendritic cells), thereby initiating and modulating adaptive immune responses (Kawai and Akira, 2007). Alternative splicing of MyD88, a key adapter for TLR signalling, is essential for regulating immune responses and preventing chronic inflammation. Alternative splicing of MyD88, a key adapter for TLR signalling, is essential for regulating immune responses and preventing chronic inflammation (Lee et al., 2024). In the future, the development of drugs targeting SRSF1, HNRNPU, or MyD88 alternative splicing processes may help to modulate excessive immune responses and provide new strategies for the treatment of chronic inflammatory diseases such as autoimmune diseases.

### 4.4 TGF-β

TGF-β, known as Transforming Growth Factor-β, is a multifunctional cytokine belonging to the TGF-β superfamily. TGF-β plays an important role in cell growth, differentiation, adhesion, migration and apoptosis, and is one of the key factors regulating cell development and physiological functions (Yang et al., 2010; Larson et al., 2020). TGF-β1 secreted by ccRCC activates P38 and induces H3 Ser10 phosphorylation through a signalling pathway, which in turn increases SRSF3 and SRSF5 expression in T cells. Inhibition of the TGF-β1 signalling pathway or SRSF3/SRSF5 expression may become a new strategy for ccRCC immunotherapy (Wu et al., 2020). TGF-β is a multifunctional cytokine that regulates multiple life processes of SRSF splicing factors through complex signalling pathways. It has a wide range of applications in clinical treatment and research, and is one of the important research directions in the future biomedical field.

### 4.5 Macrophages

When the body is infected or injured, macrophages will be activated and gather towards the site of infection or injury to perform their immune function. Depending on the activation state, macrophages can be classified into different subtypes such as M1 type and M2 type (Mehla and Singh, 2019; Anderson et al., 2021). Apoptosis is an autonomous and orderly way of cell death under genetic control, which involves a series of processes such as activation, expression, and regulation of genes, ultimately leading to cell death and removal (Fleisher, 1997).

It has been shown that SRSF6 is a guardian of mitochondrial homeostasis. Transcriptomic analysis of mouse macrophage cell lines revealed that SRSF6-dependent mitochondrial coordination is largely directed by alternative splicing of the pro-apoptotic protein BAX. Deletion of SRSF6 promotes the accumulation of Bax-k, which sensitizes macrophages to cell death and triggers the upregulation of interferon-stimulated genes via cGAS sensing of cytoplasmic mitochondrial DNA, further exacerbating the inflammatory response. Upon pathogen induction, macrophages regulate SRSF6 expression to control the release of immunogenic mitochondrial DNA and adjust the threshold for entry into programmed cell death. Thus, SRSF6 plays an important role in macrophages to balance the immune response and prevent tissue damage. Based on the critical role of SRSF6 in preventing excessive inflammatory responses, modulators targeting SRSF6 may be developed in the future to control the extent of inflammatory responses and avoid tissue damage (Wagner et al., 2022). SRSFs may also have an effect on macrophage survival and apoptosis. It was found that SRSF10 may be involved in immune evasion of tumors through the glycolytic pathway, and that targeting SRSF10 with the selective inhibitor 1c8 is able to enhance the efficacy of the monoclonal antibody against programmed cell death 1 (PD-1), suggesting that SRSF10 may play an important role in macrophage polarization and immunotherapy of hepatocellular carcinoma (Cai et al., 2024). In the future, targeted inhibitors against SRSF10 will be found to potentially inhibit M2 macrophage polarization, thereby enhancing the efficacy of anti-PD-1 hepatocellular carcinoma therapy (Cai et al., 2024). This provides new ideas for the treatment of T cell-related diseases.

## 5 Conclusion

Alternative splicing is an important mechanism for processing pre-mRNAs during gene expression, and AS determines exon exclusion and inclusion to produce mRNA and protein isoforms with completely different functions from a single transcript, suggesting that alternative splicing is an important part of gene expression. SRSF splicing factors, as key regulators in cell biology, are widely involved in the regulation of everything from RNA stability to RNA splicing and even RNA modification (m6 A), export and translation. In eukaryotic cells, alternative splicing increases the diversity of the transcriptome, SRSFs regulate almost every step of AS and are important trans-acting factors, SRSFs bind to pre-mRNA to promote splice site elimination and spliceosome assembly, which are collectively involved in the regulation of spliceosome function. Aberrant expression of SRSFs leads to aberrant splicing of RNA, generating isoforms that affect tumor cell proliferation, migration, and apoptosis resistance. Numerous studies have shown that most SRSFs are upregulated as proto-oncogenes in the expression of various tumors and are involved in the development and progression of a variety of cancers, such as pancreatic, breast, small-cell lung, prostate and colorectal cancers. In addition, SRSFs also mediate tumorigenesis by regulating alternative splicing of tumor-associated genes, providing a large number of targets for tumor control.

The immune function of the human body is closely related to the development of tumors, and when the host’s immune function is low or suppressed, the incidence of tumors increases. SRSFs affect the expression level and activity of cytokines by regulating the alternative splicing of tumor immune-related genes, which in turn affects the function of immune cells in the tumor microenvironment, especially playing an important role in the development, differentiation, function, and disease development of T cells, macrophages, and related immune cells.

In conclusion, the current study suggests that alternative splicing is an important part of gene expression and that SRSFs are widely involved in RNA metabolic events as important regulators. Abnormal activation of SRSFs has been associated with cancer, the immune system, and autoimmune diseases, etc. Molecular inhibitors are important tools in cancer therapy, and their high efficiency and specificity make them widely used in targeted therapy and immunotherapy. Future studies should further explore the molecular mechanisms of SRSF family members and develop more efficient and specific inhibitors to advance their clinical use. Although there are still many problems in the study of SRSFs, we believe that with the deeper study of SRSFs, the related challenges will eventually be overcome, and will provide new methods and ideas for the treatment of diseases.
